# Long Term Population, City Size and Climate Trends in the Fertile Crescent: A First Approximation

**DOI:** 10.1371/journal.pone.0152563

**Published:** 2016-03-28

**Authors:** Dan Lawrence, Graham Philip, Hannah Hunt, Lisa Snape-Kennedy, T. J. Wilkinson

**Affiliations:** Department of Archaeology, Durham University, Durham, United Kingdom; University at Buffalo, UNITED STATES

## Abstract

Over the last 8000 years the Fertile Crescent of the Near East has seen the emergence of urban agglomerations, small scale polities and large territorial empires, all of which had profound effects on settlement patterns. Computational approaches, including the use of remote sensing data, allow us to analyse these changes at unprecedented geographical and temporal scales. Here we employ these techniques to examine and compare long term trends in urbanisation, population and climate records. Maximum city size is used as a proxy for the intensity of urbanisation, whilst population trends are modelled from settlement densities in nine archaeological surveys conducted over the last 30 years across the region. These two measures are then compared with atmospheric moisture levels derived from multiple proxy analyses from two locations close to the study area, Soreq Cave in Israel and Lake Van in south-eastern Turkey, as well as wider literature. The earliest urban sites emerged during a period of relatively high atmospheric moisture levels and conform to a series of size thresholds. However, after the Early Bronze Age maximum urban size and population levels increase rapidly whilst atmospheric moisture declines. We argue that although the initial phase of urbanization may have been linked to climate conditions, we can see a definitive decoupling of climate and settlement patterns after 2000 BC. We relate this phenomenon to changes in socio-economic organisation and integration in large territorial empires. The complex relationships sustaining urban growth during this later period resulted in an increase in system fragility and ultimately impacted on the sustainability of cities in the long term.

## Introduction

The rise of cities forms an enduring theme in global history [1], and of the many approaches to urban analysis, city size continues to form a key dimension [2]. However, because ancient cities were social and economic phenomena [3], and interacted with their physical environment at a variety of spatial scales [4], the analysis of temporal trends in urban development can appear bewilderingly complex. Among the many forces acting on the ancient city, climate is often regarded as significant because the provisioning of such large settlements is in part related to food production and therefore rainfall and evaporation. For example, cities or city complexes have been suggested to have collapsed as a result of climate events [5, 6, 7]. However, despite the capricious nature of the environment, the relationship between societal change and climate continues to be debated. More recently, other factors such as resilience, which allows population centres and their cities to endure in a restructured form, have been adopted within alternative models to collapse [8, 9].

Here we employ a sample of ancient Near Eastern cities dated between about 6000 BC and AD 1000 using settled area as a proxy for city population and relate this to estimates of regional population and prevailing climate to examine their long-term relationship. Maximum city size in hectares has often been taken as a proxy for social complexity, at least over the long term [10], but is also of interest in its own right [11, 12]. Total settlement (in the form of aggregate site areas in hectares derived from archaeological survey) is used as a proxy measure for regional population [13] and allows us to compare across time and space. Settlement trajectories are derived from both rural and urban scale settlements which frequently demonstrate different trajectories of growth through time [14, 15]. City size and aggregate site areas are then compared with atmospheric moisture as represented by oxygen and carbon isotope data for two locations, Soreq Cave, Israel [16, 17] and Lake Van, Turkey [18, 19, 20], as well as other proxy records. The Soreq Cave and Lake Van records represent the most reliable long term sequences currently available for the region and frame the study area to the south-west and north-east respectively. The objective is to examine the degree of accord between long-term trends in climate on the one hand and the two indicators of settlement (maximum city size and total combined site areas) on the other. We find that the earliest urban sites emerged during a period of relatively high atmospheric moisture levels and conform to a series of size thresholds. However, after the Early Bronze Age urban size and population levels increase rapidly whilst atmospheric moisture declines. We argue for a definitive decoupling of climate and settlement patterns after 2000 BC and relate this to changes in socio-economic organisation and integration in large territorial empires.

## Materials and Methods

### Mapping Settlements and Cities

**Fig 1:**
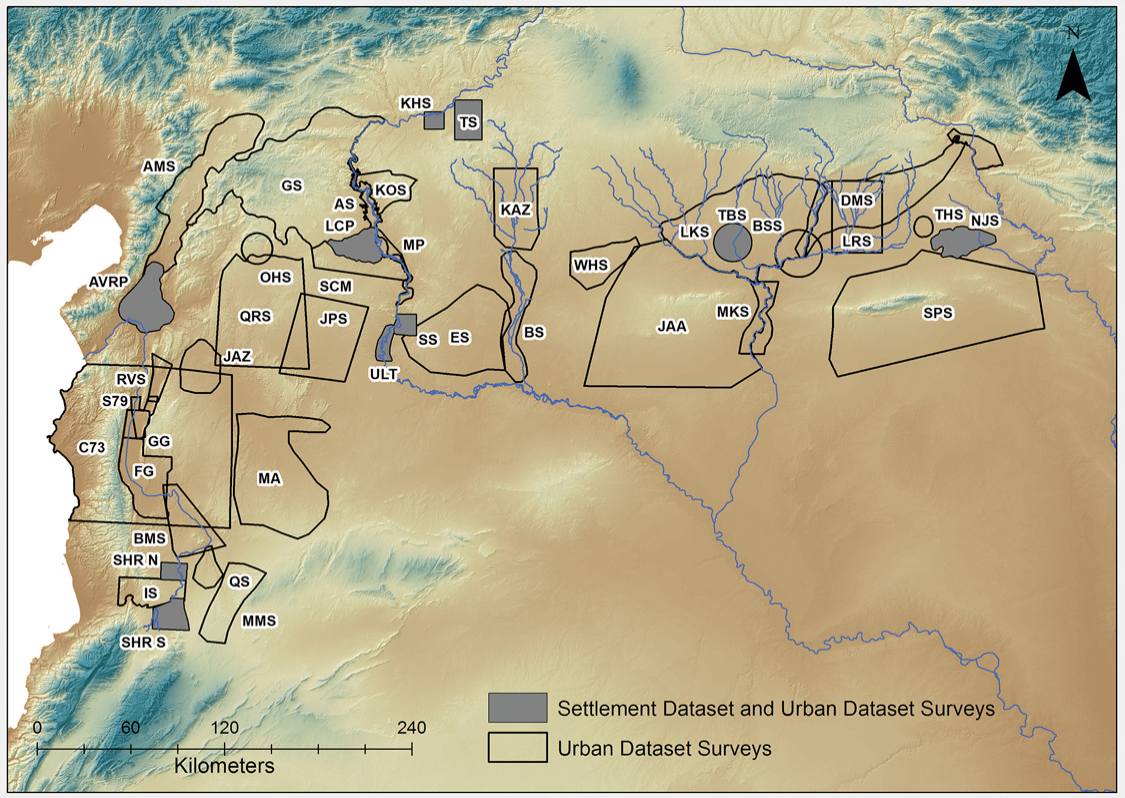
Surveys used in this paper. Shaded surveys provide sample site areas and densities, other surveys provide information for the urban dataset. Background SRTM DEM courtesy of the U.S. Geological Survey.

The pattern of ancient settlement has been established by coupling archaeological surveys with remote sensing and selected data from excavations to supply the distribution of the occupied sites, estimates of their size and periods of occupation [21, 22]. The main study region encompasses the northern Fertile Crescent, defined as the dry farming plains bounded by the mountains of the Zagros and Taurus ranges to the east and north, several coastal mountain chains including the Amanus, Jebel Ansariyah, Lebanon and Anti-Lebanon to the west, and the arid steppe zone to the south. Today this region includes much of Syria, south-eastern Turkey and northern Iraq. The study region was selected due to the significant quantity and high quality of archaeological data available, although it should be noted that far less research has been conducted in the plains of northern Iraq than in Syria and southern Turkey due to modern political circumstances. Whereas in the Near East archaeologists have relied upon single surveys to provide estimates of long-term settlement, for this first approximation data has been collated from nine surveys (Fig 1, Table 1) to supply estimates of changes in the quantity of settlement (i.e. derived from total settlement area) for the last 8000 years (S1 Appendix Sheet 1). These surveys have been selected due to similarities in research aims, methods and general approach and together provide a representative sample of total site areas in the region’s main agricultural basins [14]. Subject to the normal caveats regarding on-site population densities [23, 24, 25], aggregate site areas provide approximations of local area populations [13], especially when applied to larger numbers of settlements [26]. Here we retain the use of ‘raw’ site area so that estimates are derived from primary data rather than secondary estimates.

**Table 1:** Surveys used in this paper. Surveys in bold provide sample site areas and densities, other surveys provide information for the urban dataset. See S1 Appendix Sheet 1 for details.

Survey	Acronym
Amanus Survey	AMS
**Amuq Valley Regional Project**	**AVRP**
Balikh survey	BS
Bartl and al-Maqdissi	BMS
Brak Suburban Survey	BSS
Carcemish-Birecik	AS
Cizre-Silopli Survey	CSS
Copeland and Moore	SCM
Courtois 1973	C73
Einwag Survey	ES
Fortin Ghab	FG
Gaziantepe	GS
Graff Ghab	GG
Ibanez Survey	IS
Jabbul Plain	JPS
Jazr Plain Survey	JAZ
Jebel Abd al-Aziz	JAA
Karul and Ozdogan Survey	KOS
**Kurban Hoyuk Survey**	**KHS**
**Land of the Carcemish Project**	**LCP**
Leilan Region Survey	LRS
Lyonnet Khabur	LKS
Maqdissi Survey	MMS
Marges Arides	MA
McClellan and Porter	MP
Meijer	DMS
Middle Khabur Survey	MKS
**North Jazira Survey**	**NJS**
Oylum Hoyuk Survey	OHS
Qatna Survey	QS
Qoueiq and Rifa'at Survey	QRS
Rouj Valley	RVS
Sauer 1979	S79
**Sites and settlement in the Homs Region North**	**SHR N**
**Sites and settlement in the Homs Region South**	**SHR S**
Sinjar plain	SPS
**Sweyhat Survey**	**SS**
**Tell Beydar Survey**	**TBS**
**Tell Hamoukar Survey**	**THS**
**Titrish Survey**	**TS**
Upper Lake Tabqa Survey	ULT
Wadi Hammar Survey	WHS
Yarmdici Survey	KAZ

Estimates of urban areas were made using data from field surveys and archaeological excavations (Fig 1), supplemented by visual inspection of satellite imagery [27, 28]. For this study all sites dating between the sixth and early second millennium BC greater than 10 ha in area were analyzed for the main study region (S1 Appendix Sheet 2). The vast majority of sites during this period were between 1 and 3 hectares in size [13, 29], whilst there is a marked lack of sites of between 5 and 10 hectares in the surveys examined. We are therefore confident that sites over 10 hectares represent a different order of settlement. A second sample of data was used for post-2000 BC cities (S1 Appendix Sheet 3). The settled areas of these were derived from a combination of historical sources and the archaeological record cross checked, where possible, against CORONA imagery from the 1960s and high resolution images from 2000 and later. Because urban site size and the number of larger sites increases through time, for this initial study the size threshold for inclusion in the second sample is set at 100 hectares, corresponding to the size of the largest sites in the pre-2000 BC sample. The post-2000 BC cities are drawn from a larger spatial extent than the earlier sample because the size of the polities involved increases beyond that of the original study area. In some instances, such as the Middle- and Neo-Assyrian capitals, the urban centres remain within the northern Fertile Crescent but are situated in areas subject to far less recent research, meaning broader settlement trends are unavailable. However, we have also included the large urban capitals of later polities situated in southern Mesopotamia and Turkey because they likely drew on the resources of the northern Fertile Crescent to facilitate urban expansion.

### Estimates of temporal trends

The comparison of long term trends in archaeological settlement at a regional scale relies on the construction of a series of phases which have relatively distinct ceramic assemblages, underpinned by groups of C ^14^ dates which serve to relate the ceramically-defined periods to which survey data is usually assigned to absolute dates in years. Because changes in ceramic types are not uniform across either space or time, local sequences are developed to date sites with higher levels of precision [30, 31, 32, 33]. Comparing data at a regional level therefore requires the reintegration of these local phases into a single overarching framework. The solution applied here is to assign all ceramic phases used within individual surveys and sites start and end dates in years, drawing upon the most recent excavation data. By transforming these phases into a similar metric, we can display them graphically and model trends in site areas in a way which allows for visual comparisons to be made [34, 35]. Importantly, this process is scalable, such that we can compare individual surveys, combinations of surveys and overall settlement trends by adding together data from different groups of sites. In this study both local and regional settlement data are displayed in a series of 'time slices' of 100 years duration. The aggregate settlement figure for each time slice represents the combined area of all sites within the nine sample surveys which included ceramics securely dated to within that 100 years.

## Results

### Settlement size estimates and long-term trends

**Fig 2:**
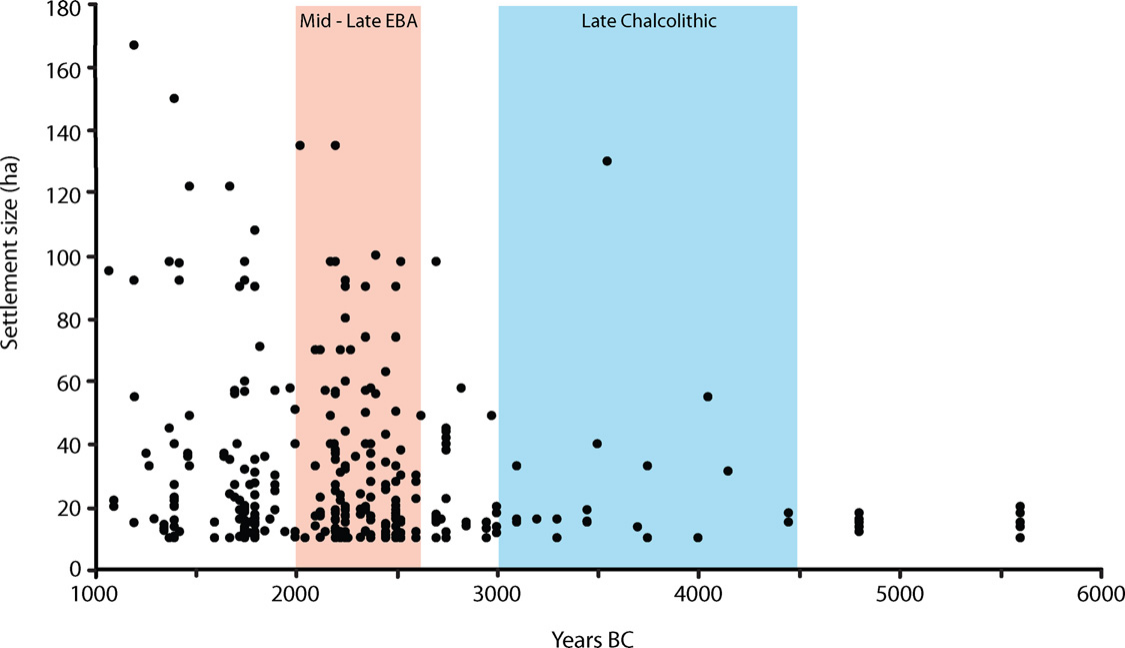
Settlement size for all recorded sites over 10 ha between 6000 BC—1000 BC for the northern Fertile Crescent. Note the maximum sizes of 20 ha (6000–4200 BC); 40–60 ha (4200–3000 BC) and ca. 100–120 ha (3000–1200 BC), but with significant "plumes" beyond these figures at intervals between 4000 and 2200 BC. Sites under 10 ha are not shown (N = 351).

The initial dataset, including all large sites dating from the sixth to the third millennia BC, captures both the first (Late Chalcolithic c. 4400–3400 BC), and second (c. 2600–2000 BC) cycles of urban civilization in the region [36]. Whereas the largest sites during the preceding Halaf and Ubaid phases do not exceed 20 ha, well-defined Late Chalcolithic centres of the initial urban cycle range up to 40–60 ha, with "plumes" (or break-outs) up to 130 or even 300 ha [27]–although the duration and density of settlement at the largest of these remains uncertain [36]. The second urban cycle comprises a large number of smaller cities of the mid third millennium BC which rarely exceed 100 ha in area but again show occasional plumes above this figure (Fig 2). The 100 to 130 ha figures apparent for sites of the period 2500–2300 BC conforms to a plateau in settlement size data for the region [37, 38] and globally for large prehistoric agrarian settlements [11].

**Fig 3:**
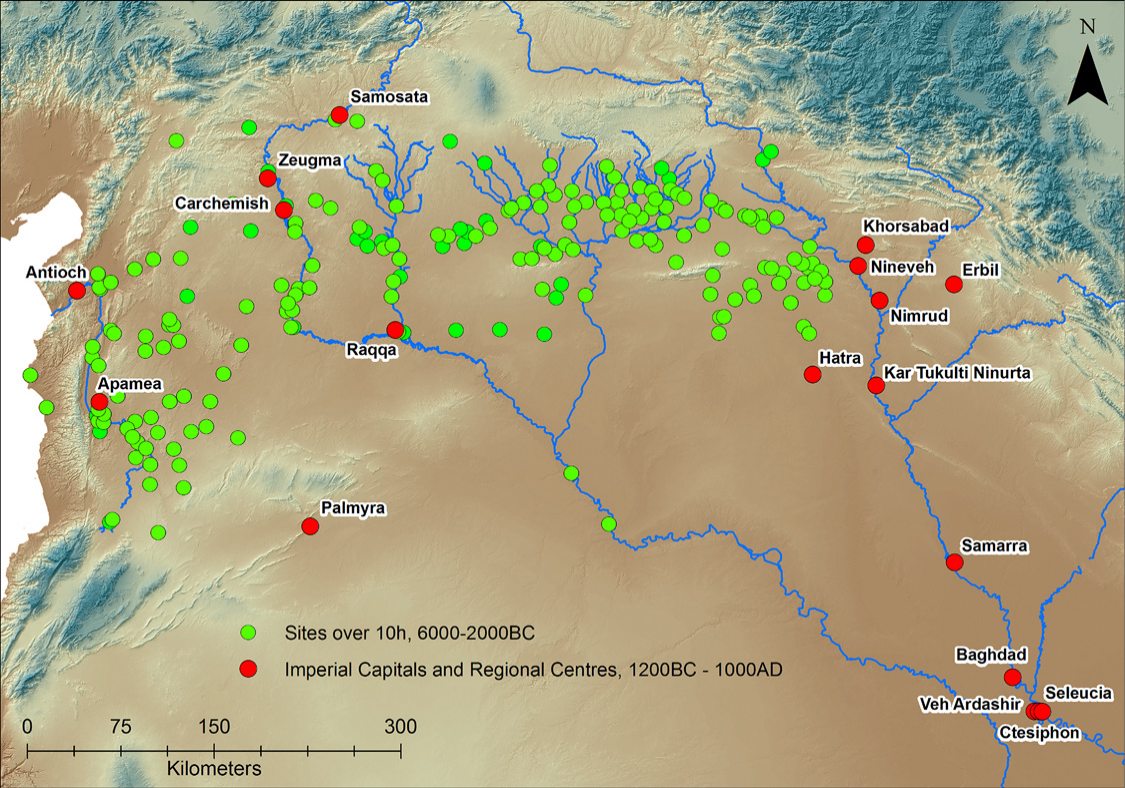
Urban Settlements used in this paper. The green circles represent all sites over 10 hectares from 6000–1200 BC, red represents the dataset of urban sites over 100 ha dating between 1200 BC and 1000 AD. Background SRTM DEM courtesy of the U.S. Geological Survey.

The second dataset is drawn from sites dated to post 2000 BC, and for simplicity we have only presented cities larger than 100 ha, that is those which transcend the site size ceiling of the earlier dataset. Initially urban site size remains relatively stable, with sites such as Qatna (120 ha) displaying a change in morphology in comparison to earlier cities but remaining within the 100–130 ha envelope. With the expansion of powerful regional polities such as the Middle Assyrian empire around 1300 BC, city size expanded rapidly, first with Kar Tukulti Ninurta (250 ha) and Erbil (330 ha), and later with several Neo-Assyrian capitals, culminating with Nineveh in the 7th century BC (750 ha). During the first millennium AD capital cities continued to grow, without apparent restraint, until the Abbasid era when Samarra and Baghdad attained areas of 4635–5800 and 4500–7000 ha respectively (Fig 3).

### Aggregate Settlement for the Northern Fertile Crescent

**Fig 4:**
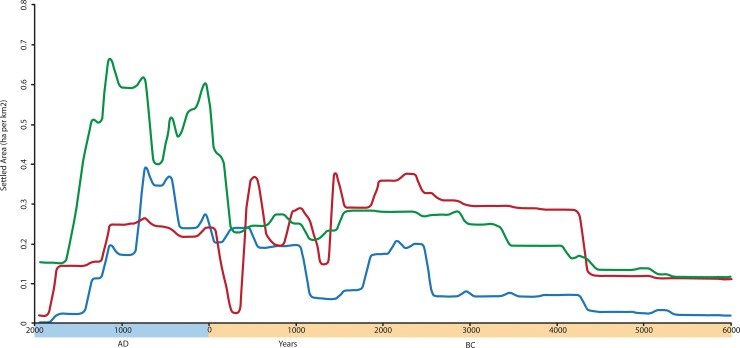
Aggregate settlement density for the three sub-regions of the northern Fertile Crescent expressed as settled area (hectares) per km^2^ surveyed. Red line represents the East (Jazira) region, blue line the Central (Euphrates Valley) region, green line the West (Orontes Valley) region.

Settlement data are expressed first for the western, central and eastern Fertile Crescent and second as a total for all areas summed together. Aggregate settlement densities for each region are important because they demonstrate that settlement trends vary regionally, in particular from west to east, and inform on the regionally specific socio-economic trends against which the development of largest sites must be understood. For example, the pronounced increase in settlement density from the 3rd century BC to the 13th century AD visible in western Syria and southern Turkey is more muted in the Middle Euphrates, and is replaced by a relative decline for these same phases further east in the Jazira (Fig 4)

When the combined settlement across all the surveys is plotted, the most obvious long-term trend is that of increased settled area (and by inference population) over time. The peaks and troughs that are apparent within the overall trend might correspond to episodes of cyclical growth and decline for long term population trends [39], but in the case of the Fertile Crescent they appear to result, at least in part, from the pulsating growth of cities which contribute to long-term settlement curves [14, 15].

### Climate Reconstruction and Temporal Scale

**Fig 5:**
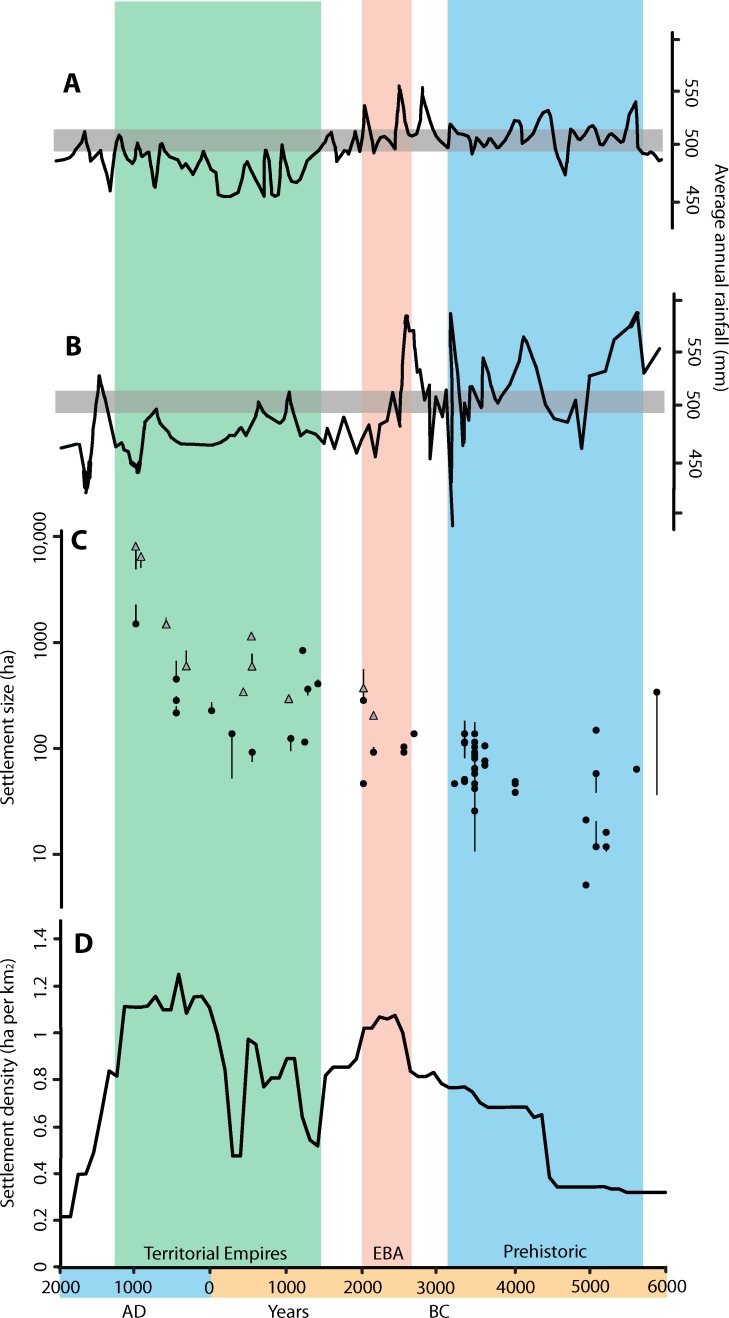
The combined records of climate, maximum city size and aggregate settlement area for the northern Fertile Crescent. A: Atmospheric moisture derived from Lake Van sequence (14), note grey line indicates modern rainfall level, B: Atmospheric moisture derived from Soreq Cave sequence (12), note grey line indicates modern rainfall level, C: Maximum size of urban places between 6000 BC and AD 1000 in hectares (N = 52). Black dots indicate sites within the main study region, grey triangles indicate sites in the wider region. Both symbols represent our own estimate of site size, bars include the total range of estimates in the literature D: Total aggregate settlement for nine sample surveys across the northern Fertile Crescent expressed as settled area (hectares) per km^2^.

Analyses of the impact of climate change on human communities over time have been conducted at a variety of temporal scales. For the Near East, the existence of a connection between relatively short term phenomena such as the Younger Dryas or the 8.2k climatic event and major changes in the organization of settlement and society is now widely accepted, while the relationship between the 4.2k event and third millennium BC settlement has been much debated [40, 41, 42]. The latter is of some interest here because it has been specifically related to dramatic declines in levels of both urban and rural settlement [36, 40]. Less has been said about long-term settlement trends in relation to climate proxy records, especially in relation to our main study region. This is in part a result of the long-term pattern of low rainfall and consequent discontinuous sediment records from lakes or marshes, as well as the paucity of suitable caves for speleotherm sampling [43]. Here we present data from a range of proxy records for long term climate fluctuations located close to and within the study area, and illustrate the two closest, the varve sequence from Lake Van in south-eastern Turkey and speleotherm evidence from Soreq Cave in Israel, in Fig 5. Lake Van is a large terminal lake in south eastern Turkey. Sediment cores taken in 1990 provide a continuous varve record for the entire period relevant to this study and have produced Mg/Ca and stable oxygen isotope data as well as pollen and charcoal data [20]. Soreq Cave contains a continuous speleotherm record from 185 ka to the present and has provided oxygen and carbon isotope records [44]. The published studies from the two records identify the following broad trends in atmospheric moisture:

- From around 5500 BC (7450 BP) for Lake Van [18, 19, 20] and at least 5000 BC for Soreq Cave [16, 17] a moister climate than today prevailed for the Chalcolithic and early phases of the EBA. This continued into a transitional period between 2300–2000 BC characterized by increased aridity and a much discussed phase of settlement devolution.
- Following this transitional phase, around 2000 BC, there was significant atmospheric drying which continued with both temporal and regional fluctuations until at least the end of the first millennium AD [44] before a slight increase in moisture to the present day.

These trends are visible in oxygen and carbon stable isotope sequences across the East Mediterranean and Arabia [45], and more locally from the Nar lake in central Turkey [46] and the Black Sea coast [47], as well as through analysis of stable isotopes alongside diatoms and pollen records from crater lakes at Eski Acıgöl, also in central Turkey [48]. Evidence from four separate pollen cores from the Dead Sea and Sea of Galilee indicates a high and sustained level of Mediterranean tree pollen, linked to higher levels of precipitation, throughout most of the 3^rd^ millennium BC, a sharp drop in the early 2^nd^ millennium and a highly variable but overall lower level from approximately 1800 to 600 BC [49]. Making allowance for the slower response of atmospheric pollen concentrations to variations in precipitation than is the case with the isotope data, the evidence of this core is broadly in line with the other indicators. Recent studies investigating climate change at a millennial scale using proxy data from within the main study region provide evidence for a similar shift in atmospheric moisture levels towards greater aridity after the Chalcolithic and Early Bronze Age periods, as well as a high degree of regional variability in climatic effects. These include analyses of stable isotopes within barley grains [50] and taxa changes in charcoal fragments [51], both recovered from archaeological contexts at sites spanning the region spatially and temporally, as well as geoarchaeological studies [52].

### Climate and Settlement Records Combined

Examining the climate and settlement records together allows us to compare trends in the long term (Fig 5). A marked divergence between regional settlement / urbanization and long-term climate appears around 2000 BC. Before this, late 5th and earlier 4th millennium BC urbanization occurred within the relatively moist phase between 5500 and 2200 BC, which was also a period of generally dense rural settlement [15]. Aggregate settlement (in occupied hectares per km^2^) then attains a peak between 2500 and 2200 BC after which the settlement devolution of the late third millennium BC occurred (Fig 5). The impact of the 4.2k event on our study area is not immediately clear in the settlement data, in part due to familiar issues of precision in ceramic dating which make it difficult to ‘see’ such short term events in the archaeological record at this scale, especially in survey evidence [34, 35]. However, a reduction in the number of urban sites securely dated to the period in which the event may have occurred is visible. This will be discussed in a future paper. After the end of the Early Bronze Age there followed a general decline in settlement during the 2nd millennium BC (MBA and LBA) with secondary declines at c. 1400 and 500 BC. These latter instances are almost certainly the result of periods of time for which chronologically-sensitive types are lacking in the ceramic record, meaning sites from this period were not recognised during the original field surveys. Between these two minor declines, the expansionist phase of the Iron Age Neo-Assyrian Empire is evident by a double peak ca. 1100–600 BC. This expansion includes the rapid growth of imperial cities of between 250 and 750 ha area, and is followed by an additional phase of settlement growth corresponding to the imperial expansion of the Seleucid, Roman, Byzantine, Sasanian and Early Islamic empires. These later phases are associated with the growth of major cities such as Antioch (400–600 ha), Ctesiphon (540–750 ha), Raqqa (1350–2064 ha) and Constantinople (1400–1555 ha), several of which lie outside the sample areas. The polities which emerged during this period were of a size that extended beyond the core area under consideration in this study, and the location of the largest sites was influenced as much by the external political decisions of imperial elites, as environmental factors. Such growth culminated in the massive urbanisation of the Early Islamic cities of Samarra and Baghdad. Although these urban expansions are not necessarily comparable, for example the mega sites of the Abbasid period, specifically Samarra, include huge areas allocated to palaces and military compounds [53, 54], they demonstrate eloquently that imperial urban growth consistently outstripped earlier phases. Finally, a poorly resolved phase of decline after 1000 AD corresponds to the Middle Islamic period and the Mongol invasions when, in addition to a lack of settlement data, there was an absolute decline as well as a fragmentation of the earlier Islamic empires. Despite these medium-term fluctuations, which might be understood in terms of Braudel’s *conjunctures*, the trend over the *longue durée* is one of a general increase in scale of urban settlement throughout most of the Holocene.

We argue that both the overall scale of settlement and the size of the largest individual units became definitively de-coupled from climate after 2000 BC (i.e. post 3950 BP) with the result that the trends of settlement and urbanisation in the Iron Age and Classical-Islamic periods show no obvious relation to atmospheric moisture. Settlements of the Chalcolithic developed within more propitious climatic conditions, such that urbanisation between 4400 and 3400 BC may have been linked to production surpluses related to higher rainfall. We cannot be certain that the climate was a causal factor in this development, or that similar sites would not have arisen in drier conditions, but there is an initial correlation between the two datasets which becomes much less clear during and after the secondary phase of urbanism from 2600–2000 BC. After 2000 BC both rural settlement and city growth occurred during phases of generally drier conditions, albeit with regional variations [50] and after 1200 BC we witness the growth of cities that were far beyond the size that could have been supported by rain-fed agriculture in their immediate vicinity.

Maximum city size increased with aggregate settlement area, which is taken to be proportional to total population (Fig 5). This supports the suggestion that overall wealth is proportional to the number of people working [55, 56]. However, over time city size greatly outstrips rural settlement growth, suggesting the economy of major ancient cities is functionally related not only to production surpluses generated from their hinterlands, but also to systems of taxation, type of labour (slaves, tenants or free) as well as broader transport networks and long-distance trade. The increasing reliance of these expanding urban sites on specific forms of political and economic integration had profound implications for their long term sustainability. Nevertheless, regional population and urbanization both follow similar trends, with peaks in urban development during the mid-third millennium (EBA), the early first millennium BC (Neo-Assyrian) and first millennium AD (Roman—Early Islamic) corresponding to peaks in aggregate settlement area (Fig 5).

After 2000 BC, the relationship between atmospheric moisture and both aggregate settlement and maximum city size is weak, with both the Soreq Cave and Lake Van data indicating relatively dry conditions, and although occasional moister episodes are apparent (as in the late 1st millennium BC at Soreq) they do not correspond to peaks in settlement (total site area) or urbanization. Moreover, regional variations in climate become significant with, for example, increased rainfall in western Anatolia between 0 AD and 500 AD [57, 58] and decreased moisture at Soreq between 100 AD– 700 AD [44, 57]. Although events such as the dust veil of 536 AD [59, 60] may have ushered in particularly cool years, urbanization and population size continued upwards into the 9th century AD. Similarly, brief episodes of increased moisture have been episodically recorded [57, 61] but none of the proxy records shows a consistent trend of increasing moisture that could account for the growth of settlement and cities in the first millennium AD. In other words, although rainfall variations may contribute to episodic shortfalls in agricultural production, the continuing increases in population and maximum city size are evidently responding to the systems of provisioning and organisation which emerged in the later empires.

## Discussion

The routine expansion of cities beyond the 120–130 ha ceiling occurs from around 1300 BC, during the Middle and Neo-Assyrian empires, both of which maintained a relatively consistent sphere of control extending from the Zagros Mountains to the Euphrates River for some six centuries. The later settlement and urbanization peak corresponds to an increase in economic activity seen in the Mediterranean and Classical world, which saw "one of the strongest economic efflorescenses in pre-modern history" [56] and a highly monetized economy, especially until ca. 200 AD. In the case of the Near East, the initial prime players were the Neo-Assyrian and Achaemenid empires (in the first millennium BC), with this broad growth continuing to the apogee of the Islamic period in the 9th century AD.

During this lengthy phase of imperial expansion, from 1300 BC to ca. 1200 AD, capital cities were moved frequently according to the motivations of elites, and urban growth was fuelled by the incorporation of ever larger areas of cultivable land. Cities were supplied with agricultural products, often farmed intensively, via dense scatters of rural settlement, frequently inhabited by deported populations, or by increased areas of irrigated land which extended as a result of capital-intensive irrigation systems. This technology, in the form of major irrigation systems dug throughout much of the former rain-fed lands [62, 63, 64], started in the Middle Assyrian period, gained momentum in the Neo-Assyrian Empire and became increasingly important during the Roman, Sasanian and Islamic periods. The proliferation of irrigation systems provided more reliable yields for taxation and the state revenues, and overcame the disadvantages of earlier less efficient practices of dry-farming and the resulting uneven taxation; expanding the tax base also increased revenues for further state expansion [65]. The increase in the scale and power of polities from the Middle Assyrian period onwards (i.e. after ca. 1200 BC) provided an "administrative umbrella" which allowed for the extension of large irrigation systems often into multiple catchments. Enlarged polities facilitated technology transfer over larger areas, which by the Classical and Early Islamic periods contributed to a "Green Revolution" (now extended over some 1200 years from the Persian and Hellenistic to Early Islamic times [66, 67]) and ushered in a suite of novel orchard crops from further east, all of which required increased irrigation.

## Conclusions

The limited correlation between trends in settlement and urbanization and those of climate after 2000 BC does not mean that climate was insignificant, only that explanations need to allow for the added complexities associated with later imperial societies that included multifaceted strategies of settlement organisation, agricultural intensification, irrigation, transport (overland and via water) and taxation. From the third millennium BC onwards, much urbanisation in the Fertile Crescent was pulsating and cities were relatively short-lived, with maximum city size being maintained for only a few centuries, or even less than a century as in the case of 9th century AD Samarra [52]. Consequently, "collapse" should be understood within such episodic phases of city growth. Whereas imperial or dynastic cycles show a rise, peak and ultimately collapse, perhaps due to cycles of growth and decline as argued by Ibn Khaldun [68], decreasing returns to complexity as laid out by Tainter [69] or demographic pressures [39], maximum urban size proceeded ever upwards, with each subsequent phase of urbanization accommodating larger areas. This suggests that the size of capital cities may be related to the size and power of the polities with which they were associated, and as those polities became larger the capital city, or major regional centres such as Antioch, were able to accommodate the growing number of non-food producers that the increasing organizational complexity of these formations required. In addition, factors such as the shifting role of individual cities as military bases and their role as centres of both technological and artistic innovation increased their vulnerability to realignments of military priorities and shifts in patterns of trade or creativity.

Maximum urban size in the Early Islamic period, when cities were up to 7000 hectares and held populations of at least 280,000 people, occurred during a relative dry phase of the Holocene, when food production was able to expand as a result of increased use of canals (and other water technology) for irrigation, as well as increased transport of bulk food products by water. This not only enabled the massive growth of cities, but also permitted production to be shifted from place to place as centres of government and economy were re-located (e.g. from Samarra to Baghdad [53]). However, such expansion appears to have come at a cost. Increased tax burdens, especially to supply the army, rapid dynastic succession and political fragmentation together resulted in problems of provisioning of food and revenue delivery to the capital cities. Further decline was precipitated by the Mongol invasions which, although perhaps over-stated as a cause, devastated cities and countryside over large parts of the Middle East already weakened by over-extension during the Islamic period. The relationship between socio-economic complexity and system fragility, especially in relation to the maintenance of infrastructures through periods of dynastic upheaval and social unrest, must also have contributed to the instability of a system of accelerated urban growth that had endured for some 3000 years.

## Supporting Information

S1 Appendix Sheet 1. Total Settled Area by 100 year time slice for nine sample surveys from 6000 B.C. to present day. Sheet 2. Estimated settlement size for all sites in the study area over 10 hectares occupied between 6000 BC and 2000 BC. Sheet 3. Estimated settlement size for all sites in the study area over 100 hectares occupied between 2000 BC and 1000 AD.
(XLSX)
