# Correction: Long Term Population, City Size and Climate Trends in the Fertile Crescent: A First Approximation

**DOI:** 10.1371/journal.pone.0157863

**Published:** 2016-06-14

**Authors:** Dan Lawrence, Graham Philip, Hannah Hunt, Lisa Snape-Kennedy, T. J. Wilkinson

The following text was incorrectly omitted from the Acknowledgments: Our co-author Tony J. Wilkinson passed away during the latter stages of preparing this paper. Tony's work transformed landscape archaeology in the Middle East, and he will also be remembered by those who knew him as an unfailingly kind and generous colleague, teacher and friend. We dedicate this article to his memory.
